# Short-term responses of the anammox process to Ni(II): nitrogen removal, mechanisms and inhibition recovery

**DOI:** 10.1038/s41598-022-16566-9

**Published:** 2022-07-22

**Authors:** Yu-Qi Li, Bai-Hang Zhao, Qi Sun, Jing Zhang, Yu-Qing Zhang, Jun Li

**Affiliations:** Department of Municipal Engineering, Beijing University of Technology, Beijing, 100124 People’s Republic of China

**Keywords:** Biological techniques, Biotechnology, Environmental sciences

## Abstract

Anaerobic ammonia oxidizing (anammox) has already been recognized as an innovative and economical nitrogen removal technology. However, the effect of heavy metals on anammox bacteria in aquatic ecosystem remains largely unknown. Ni(II) is a common kind of heavy metals detected in industrial wastewater and municipal sewage treatment plants. Hence, the responses of the anammox process to Ni(II) were studied here. The results showed that anammox was the dominant reaction with Ni(II) concentrations no more than 25 mg/L. 1 mg/L of Ni(II) addition promoted nitrogen removal by anammox. The higher the Ni(II) concentrations and longer exposure time, the more inhibition for anammox bacteria was gotten. The IC_50_ of Ni(II) to anammox was determined as 83.86 mg/L by an exponential regression equation. The inhibition of Ni(II) on anammox activity was mainly attributed to intracellular accumulation Ni(II) inhibition to HDH activity. Two times increase of IC_50_ after 4 times circles of domestication suggests multiple intermittent domestication can increase the tolerance of anammox bacteria to Ni(II). EDTA washing can eliminate the inhibition of anammox activity by Ni(II) with Ni(II) addition no more than 25 mg/L.

## Introduction

Anaerobic ammonia oxidizing (anammox) bacteria can use nitrite nitrogen (NO_2_^−^-N) as an electron acceptor to directly convert ammonia nitrogen (NH_4_^+^-N) into nitrogen under anaerobic conditions^1^. Compared with traditional processes of nitrogen removal, the anammox process is more environmentally friendly and economical, making it suitable for wastewater treatment with high ammonia. However, anammox bacteria have a low growth rate (0.065–0.33 day^−1^)^2^ and are sensitive to various environmental perturbations, e.g., metals^3,4^, antibiotics^5,6^, dissolved oxygen^7,8^ and sulfides^9^. Thus, the application of anammox bacteria in wastewater treatment is limited. Some high ammonia nitrogen wastewaters^10^, such as, landfill leachate, metal smelting wastewater, and new energy production wastewater, often contain high concentrations of heavy metals (Cu(II), Zn(II), Pb(II), Ni(II), Co(II), or Mo(II)^11–13^. These metals may seriously inhibit the nitrogen removal performance of anammox sludges by interference with anammox bacteria activity^14,15^. Therefore, it is necessary to clarify the effect of metals on the anammox process.

Many studies have explored the effects of heavy metals during various nitrogen removal processes. As an important cofactor of metalloproteinases and some enzymes, Ni(II) plays an important role in the growth and metabolism of microorganisms^16^. However, excessive heavy metals combined with enzymes are inhibitory or even toxic to biochemical reactions and microorganisms, and cause the disruption of enzymatic structure and activities^17,18^. The concentration of Ni(II) in industrial wastewater and municipal sewage treatment plants typically ranges from 0.1 to 1000 mg/L^19,20^. The Ni(II) concentrations in some wastewater are much higher than the concentration required for microbial life activities. Therefore, it is important to study the effect of Ni(II) on the inhibition threshold in anammox process for anammox application in practical sewage treatment process.

Gutwinski et al.^3^ investigated the effects of different mixed heavy metals on the anammox process performance during long-term experiments, and pointed out the mixture of Zn(II), Cu(II), and Ni(II) at concentrations of 0.8, 0.075, and 0.04 mg/L caused a rapid inhibition in the anammox process. Wu et al.^21^ found an anammox system could maintain a superior performance at 10 mg/L Ni(II) during a long operation, and Ni(II) had a greater impact on the microbial community composition. The IC_50_ of Ni(II) on anammox bacteria was determined as 48.6 mg/L in batch experiments^22^. The IC_50_ values of Ni(II) for the anammox bacteria were different in reported articles due to the difference of bioreactors and operation condition. Existing reports about the effects of Ni(II) on anammox are most focus on single Ni(II) or mixed heavy metal on the inhibition phenomenon and inhibition degree about anammox activity. The inhibition mechanism is limited known. And also, less information can be found about adaptability and domestication of anammox under Ni(II).

Hence, the main objectives of this study: (1) determine the dominant bioreaction of the batch experiments under Ni(II) shock; (2) investigate the effects of Ni(II) concentration and exposure time on nitrogen removal by anammox; (3) analyze Ni(II) inhibition mechanism according to the anammox activity under Ni(II) shock; and (4) explore the cumulative inhibitory effect of Ni(II) and recovery of anammox activity of anammox sludges with different Ni(II) concentration.

## Materials and methods

### Anammox sludge and synthetic wastewater

The sludge used in batch experiments was obtained from a laboratory-scale up-flow anaerobic sludge blanket (UASB) reactor with a nitrogen load of approximately 0.5 kg-N/g-VSS/h. Before batch experiments, the sludge was washed three times using a buffer solution (pH 7.0 and 0.1 M NaCl) to remove residual matrix. Results of high-throughput pyrosequencing showed the dominating bacterial groups in the anammox sludge was the class *Planctomycetia* (48.44%). The compositions of the synthetic wastewater used in our batch experiments were listed in Table 1. NH_4_^+^-N and NO_2_^−^-N in the synthetic wastewater were provided by NH_4_Cl and NaNO_2_, respectively. According to the anammox reaction equation, the ratio of NH_4_^+^-N to NO_2_^−^-N was 1:1.32. Ni(II) was provided in the form of NiCl_2_·6H_2_O.

**Table 1:** The compositions of the synthetic wastewater used in our experiments.

Compositions	Concentration (mg/L)
NaHCO_3_	1000
KH_2_PO_4_	27.2
MgSO_4_·7H_2_O	200
CaCl_2_	300
FeCl_2_·4H_2_O	1.5
ZnSO_4_·7H_2_O	0.1
MnCl_2_·4H_2_O	0.03
H_3_BO_4_	0.3
CoCl_2_·6H_2_O	0.2
CuCl_2_·2H_2_O	0.01
NaMoO_4_·2H_2_O	0.03
NH_4_^+^-N	Add as need
NO_2_^−^-N	Add as need

### Batch experiments

Batch experiments were performed in glass vials with a total volume of 250 mL and a working volume of 180 mL. The inoculated wet sludge in each vial was 20 mL. The initial pH of reaction systems in glass vials was adjusted to 7.0 ± 0.2 using 0.1 M HCl or 0.1 M NaOH. These systems were needed to purge with high purity nitrogen for 5 min to remove oxygen. After then, the glass vials were quickly sealed with a rubber stopper and aluminum crimp. Finally, the glass vials were placed in a constant temperature shaking incubator with 35 ± 1 °C and 120 rpm. Samples in the glass vials were collected using a syringe.

#### Effects of Ni(II) concentration on anammox

The concentration of NH_4_^+^-N and NO_2_^−^-N in batch experiments were 50 and 66 mg/L, respectively. Ni(II) concentration was set as 0, 1, 2.5, 5, 10, 25, 50, 75, and 100 mg/L, respectively. Each batch experiment was carried out for 24 h. After that time, the NH_4_^+^-N and NO_2_^−^-N concentration in the glass vials basically unchanged. At the end of each batch experiment, sludge samples were retained to measure mixed liquor volatile suspended solids (MLVSS), hydrazine dehydrogenase (HDH) activity and distribution of Ni(II).

#### Effects of exposure time on anammox

The effect of exposure time of anammox bacteria to Ni(II) on the nitrogen removal and the anammox recovery was studied here. The initial concentrations of NH_4_^+^-N and NO_2_^−^-N were also 50 and 66 mg/L, respectively. According to the results of the effects of Ni(II) concentration on anammox, Ni(II) concentration in this experiment was 50 mg/L. Exposure time were set as 0 h, 12 h, 24 h and 48 h, respectively. The sludges exposed to 50 mg/L of Ni(II) concentration with different exposure time were washed with a buffer solution (pH 7.0 and 0.1 M NaCl) according to a modified EDTA wash procedure (1 mM EDTA)^23^ and fresh substrate was added to assess the anammox recovery performance.

#### Cumulative effect experiments

Two different Ni(II) concentrations, 25 and 75 mg/L, were applied in batch experiments to investigate Ni(II) cumulative effect on anammox. Four different cycles were carried out here. Each cycle was about 24 h, including 12 h experimental phase and 12 h intermittent phase. The initial concentration of NH_4_^+^-N and NO_2_^−^-N were 50 and 66 mg/L, respectively. Liquid samples were taken every 3 h to analyze nitrogen components concentration.

#### Recovery experiments about anammox activity

The sludges exposed to different Ni(II) concentration in “Effects of Ni(II) concentration on anammox” section were washed using a modified EDTA wash procedure as the same as that in “Effects of exposure time on anammox” section. Fresh substrate without Ni(II) addition was added to assess the anammox recovery performance after the EDTA washing.

All above experiments were performed in parallel three times, and the average values of the three times experiments were used to draw diagrams and tables.

### Analytical methods

NH_4_^+^-N, NO_2_^−^-N, NO_3_^−^-N and MLVSS were determined by standard methods^24^. Other indicators were determined as described in separate sections below.

#### Ni(II) determination

Two same anammox sludges (1 g wet weight) were taken from each bottle at the end of batch experiments. The samples used to measure intracellular Ni(II) concentration were washed with a modified EDTA washing procedure^25^ to remove Ni(II) adsorbed on sludge surface. The other was not treated by EDTA washing. With or without EDTA-treated sludge sample was all dissolved in a 4 M nitric acid solution with incubation at 120 rpm for 25 min. Then, the mixture was centrifuged at 12,000 rpm for 15 min to obtain a supernatant. The supernatant was filtrated through 0.2 μm membrane. Then, filtrated supernatant was used to determined Ni(II) concentration using an ICP Instrument Perkin Elmer OES Optima 2000 DV. Ni(II) from EDTA-treated sludge was about intracellular Ni(II). Extracellular Ni(II) was calculated according the subtraction result of untreated sludge by EDTA washing and EDTA-treated sludge.

Liquid samples in batch reactors were also needed to centrifuged at 12,000 rpm for 15 min and filtrated through 0.2 μm membrane to measure Ni(II) concentration in liquid.

#### Measurement of HDH activity

In order to measure HDH activity of anammox cells, 5 g (wet weight) anammox sludge were taken from each bottle at the end of each experiment, and then centrifuged at the condition of 12,000 rpm, 4 °C for 20 min. The centrifuged sludge samples were washed twice times using sodium phosphate buffer (20 mM, pH 7.0). The washed sludge samples were resuspended in 20 mL buffer to frozen (− 20 °C) for 24 h, and then sonicated (225 W, 4 °C; Ultrasonic processor CPX 750, USA) for 30 min. The supernatants (− 20 °C) were centrifuged at 12,000 rpm for 30 min and used to analyze HDH activity. HDH activity was measured by spectrophotometry at the wavelength of 550 nm and expressed as the rate of production of reduced cytochrome c (μmol cytochrome reduced/min/mg protein)^26,27^.

### Mathematical calculation

#### Determination of specific anammox activity

The specific anammox activity (SAA) was calculated by the following equation (Eq. 1).

1 $${\text{SAA}} = \frac{{\left( {{\text{C}}_{{{\text{inf}}}} - {\text{C}}_{{{\text{eff}}}} } \right) \times V}}{{24\,{\text{h}} \times {\text{MLVSS}}}}$$

where C_inf_ or C_eff_ is the total nitrogen concentrations (mg/L) at the beginning or the end of our experiments, respectively; V is the effective volume of the serum bottle (mL); MLVSS is the sludge concentration in the serum bottles (g/L).

#### Determination of IC_50_

An exponential regression equation (Eq. 2) was used to describe Ni(II) inhibition on nitrogen removal by anammox.

2 $$IP(\% ) = 100 \times \left( {1{ - }\frac{1}{{1{ + }\left( {\frac{{{\text{C}}_{{{\text{Ni}}}} }}{{IC_{50} }}} \right)^{{\text{b}}} }}} \right)$$

where IP is the inhibition percentage of anammox activity; C_Ni_ is Ni(II) concentration in the serum bottles (mg/L); IC_50_ is the inhibition coefficient with an 50% inhibition of anammox activity; and b is a fitting parameter.

#### Biological toxicity model

In order to study the inhibitory mechanisms of Ni(II) to anammox, the inhibition form of the Monod equation was used as below,

3 $$IP(\% ) = \frac{{{\text{I}} P_{{m{\text{ax}}}} C_{{N{\text{i}}}} }}{{K_{Ni} + C_{{N{\text{i}}}} }}$$

where IP_max_ is the maximum inhibition percentage of anammox activity (100%); C_Ni_ is the intracellular Ni(II) concentration (mg/gVSS); and K_Ni_ is the inhibition coefficient (mg/gVSS) associated with an 50% inhibition of anammox activity.

## Results and discussion

### Dominant reaction of the anammox sludges under Ni(II) shock

**Figure 1:**
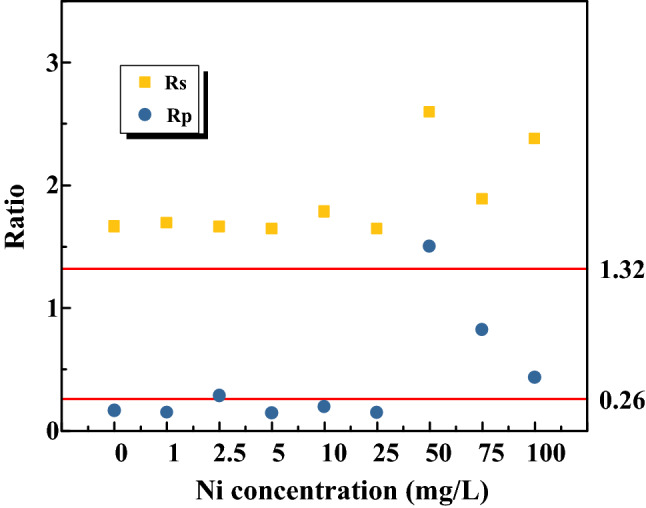
The Rs and Rp values with different Ni(II) addition.

According to the stoichiometric equation of the anammox bioreaction process proposed by Strous et al.^28^, the ratio of NO_2_^−^-N consumed and NH_4_^+^-N consumed (Rs) is 1.32, and the ratio of NO_3_^−^-N produced and NH_4_^+^-N consumed (Rp) is 0.26 in the anammox process. The two stoichiometric ratios are usually applied to indirectly judge whether anammox reaction was the main reaction in biological nitrogen removal systems^29^. Figure 1 described the change of Rs and Rp values in our experiments with Ni(II) addition. As shown in Fig. 1, the values of R_S_ and R_P_ were relatively stable and close to these stoichiometric values (1.32 and 0.26) when Ni(II) concentration was no more than 25 mg/L. However, the values of R_S_ and R_P_ fluctuated sharply with a further increasing of Ni(II) concentration. It suggested that the anammox reaction was the main bioreaction for nitrogen removal in the systems with Ni(II) addition no more than 25 mg/L. The average values of Rs and Rp were about 1.68 and 0.181 with the Ni(II) concentration no more than 25 mg/L, respectively.

**Figure 2:**
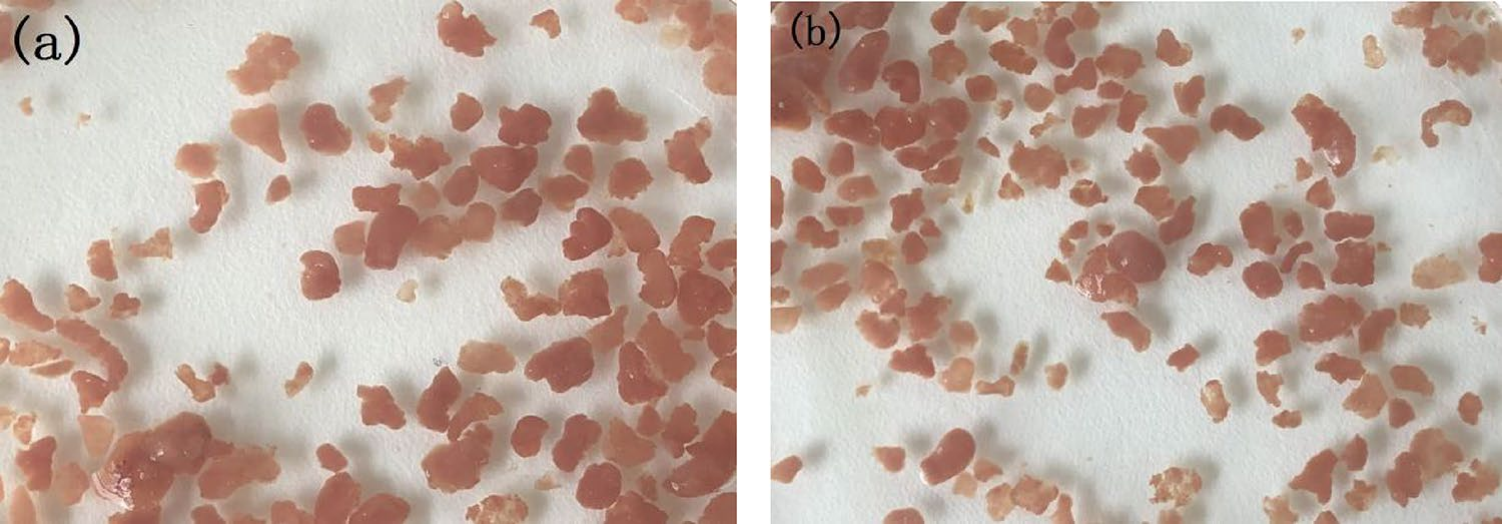
The morphology of anammox granular sludges at the end of experiments with different Ni(II) addition: 0 mg/L (**a**) and 25 mg/L (**b**).

The Rs value of 1.68 in our experiment was a little higher than the stoichiometric Rs value of 1.32. The Rp value of 0.181 was a little lower than the stoichiometric Rp value of 0.26. As is well known, microorganisms in wastewater biological treatment system are mixed flora. This resulted in the excessive removal of NO_2_^−^-N and the higher value of Rs. Special deaeration measures were not used in our experiment and the presence of nitrifying bacteria can convert NO_2_^−^-N oxide into NO_3_^−^-N. This resulted in the excessive removal of NO_2_^−^-N and the higher value of Rs. The lowed Rp value was because part of nitrate was used by denitrifying bacteria in our experiment systems. The values of Rs and Rp with Ni(II) addition equal or higher than 50 mg/L were seriously larger than those stoichiometric values. The anammox bacteria were sensitive to external environment Ni(II) addition with high concentration. Other studies have also reported that Rs and Rp ratios were not strictly consistent with these stoichiometric ratios in the anammox process^30–32^. As shown in Fig. 2, the anammox sludge kept blood-red at the end of experiments in the systems with Ni(II) addition no more than 25 mg/L, which is a significant feature of the anammox reaction. The values of R_S_ and R_P_ were close to the theoretical value of anammox reaction, which suggested that anammox reaction was also the dominant reaction in experiment with Ni(II) addition no more than 25 mg/L.

### Effect of Ni(II) on the anammox process

#### Effect of Ni(II) concentration on the anammox process

**Figure 3:**
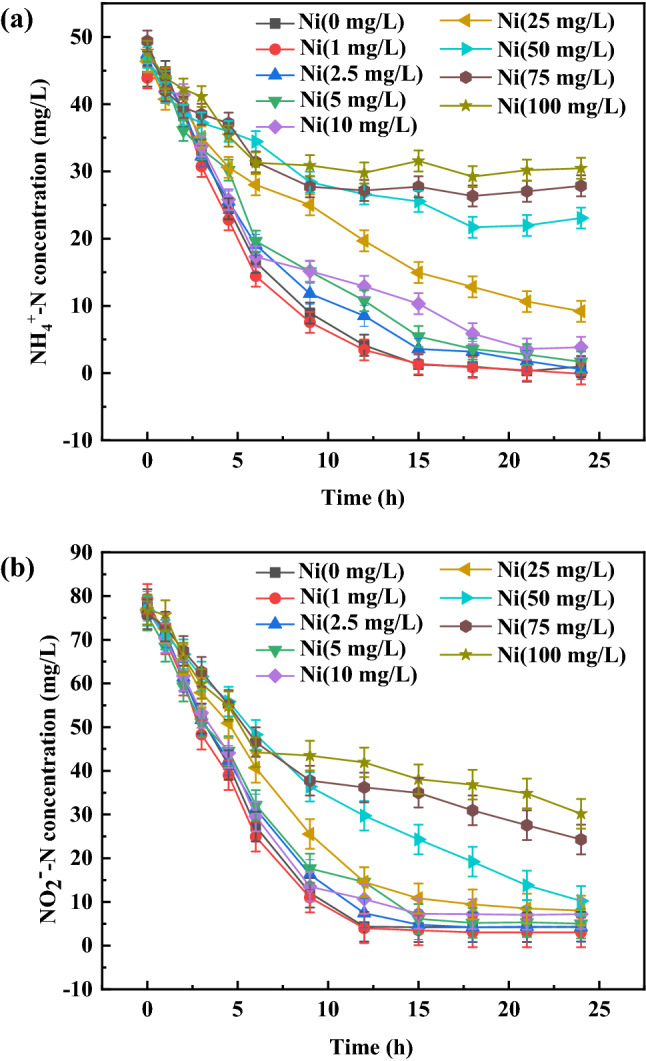
NH_4_^+^-N (**a**) and NO_2_^−^-N (**b**) concentration change with time under different Ni(II) concentrations.

The changes of NH_4_^+^-N and NO_2_^−^-N concentrations with time under different Ni(II) concentrations were shown in Fig. 3. When the Ni(II) concentration was lower than 50 mg/L, the NH_4_^+^-N and NO_2_^−^-N concentration stabilized at about 14 h with an 83%-99% removal efficiency of NH_4_^+^-N and a 94%-95% removal efficiency of NO_2_^−^-N. When Ni(II) concentration was equal or higher than 50 mg/L, the NH_4_^+^-N and NO_2_^−^-N concentration reach steady state at about 9 h. This stabilized time was shorten by 5 h than that with Ni(II) addition lower than 50 mg/L. It may be because anammox activity was quickly inhibited by Ni(II) addition with higher than 50 mg/L concentrations. This resulted in NH_4_^+^-N and NO_2_^−^-N cannot be continue removed in higher Ni(II) concentrations, and the stabilized time arrived earlier. Similar phenomena was also observed by Yang et al.^12^, who found out the stabilized time with Cu(II) concentration higher than 25 mg/L was shortened by one hour compared with that with 10 mg/L Cu(II) addition in an anammox reactor. When Ni(II) concentration equal or higher than 50 mg/L, the removal efficiency of NH_4_^+^-N and NO_2_^−^-N were 36%-50% and 60%-86%, respectively. Comparing with low Ni(II) concentrations, high Ni(II) concentrations had a more critical inhibition for nitrogen removal performance by anammox bacterial. The difference of inhibition degree between NH_4_^+^-N and NO_2_^−^-N removal efficiency was obvious under high Ni(II) addition.

An interesting phenomena was found in the system with 1 mg/L Ni(II) addition. When 1 mg/L of Ni(II) was added to the system, the concentrations of NH_4_^+^-N and NO_2_^−^-N at the ending of reaction were 0.11 mg/L and 3.04 mg/L, respectively. The concentrations of NH_4_^+^-N and NO_2_^−^-N without Ni(II) addition were 0.98 mg/L and 4.34 mg/L, respectively. The removal efficiencies of NH_4_^+^-N and NO_2_^−^-N with 1 mg/L Ni(II) addition increased by 8.75% and 5.84% than that without Ni(II) addition, respectively. It exposes that Ni(II) addition of 1 mg/L can certainly promote the activity of anammox bacteria and was beneficial for the removal of NH_4_^+^-N and NO_2_^−^-N. As an important cofactor of metalloproteinases and some enzymes, Ni(II) plays an important role in the growth and metabolism of microorganisms^16^. Chen et al.^25^ reported the anammox activity was improved at Ni(II) concentrations below 1.74 mg/L. The addition of Ni(II) with appropriate concentrations promoted the synthesis of dehydrogenation enzymes that catalyze anammox reactions and stimulate the activity of the key enzyme^15^. Hence, a higher anammox activity was gotten with 1 mg/L Ni(II) addition in our experiment.

To further quantify the inhibitory effect of Ni(II) concentration on the nitrogen removal performance by anammox bacteria, specific anammox activity (SAA) and Ni(II) concentration were fitted using an exponential regression model, as described in Table 2. SAA was calculated according to the (Eq. 1). The high value of the fitting correlation coefficient (R^2^ = 0.9974) indicated that the exponential regression model was suitable to describe the relationship between SAA and Ni(II) concentration. An 83.86 mg/L value of IC_50_ was gotten by the exponential regression model.

**Table 2:** Regression analysis of Ni(II) inhibition equations.

Model	Regression equation	R^2^
Exponential regression	$IP = 100 \times \left( {1 - \frac{1}{{1 + \left( {\frac{{C_{{N{\text{i}}}} }}{83.86}} \right)^{2.0235} }}} \right)$	0.9974
Intracellular Ni(II) and inhibition fitting	${\text{IP}} = \frac{{0.8604M_{Ni} }}{{0.07233 + M_{Ni} }}$	0.9850

#### Effect of exposure time on anammox activity

**Figure 4:**
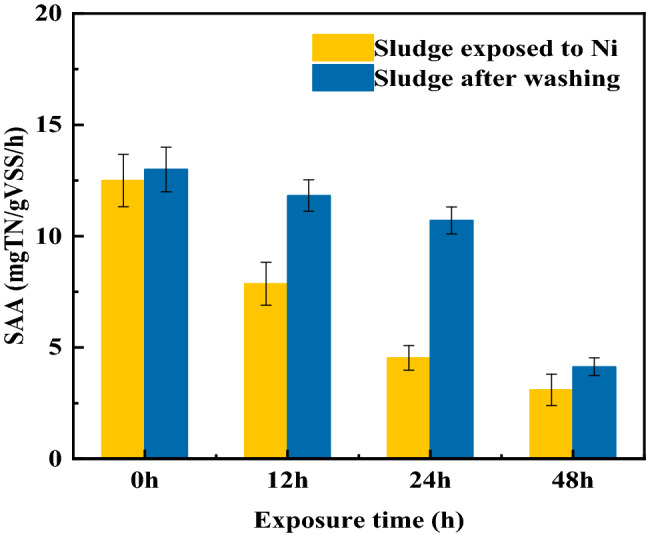
The effect of exposure time on SAA.

The effect of exposure time of anammox bacteria to Ni(II) on anammox activity was shown in Fig. 4. The SAA decreased from 11.19 mg-TN/g-VSS/h to 7.86 mg-TN/g-VSS/h with the increase of exposure time from 0 to 12 h. Extending of the exposure time to 48 h, the SAA further decreased to 3.09 mg-TN/g-VSS/h. The stronger inhibition of Ni(II) on anammox activity was existed with the more exposure time.

The recovery performance of anammox bacteria after exposing to Ni(II) were also studied here. The SAA of exposed sludge under different exposure time were all improved after EDTA washing (Fig. 4). After EDTA washing, the SAA with 12 h exposure time and with 24 h exposure time recovered from 7.60 to 12.1 mg-TN/g-VSS/h and from 4.53 to 10.7 mg-TN/g-VSS/h. The SAA with 0 h exposure time was about 11.2 mg-TN/g-VSS/h. The anammox bacteria with exposure time no more than 24 h basically restored the anammox ability that without contacted with Ni(II). When the exposure time extended to 48 h, there was a tiny digital difference between the SAA values with or without washing. Therefore, prolonging the exposure time of sludge to Ni(II) caused particularly serious inhibition of Ni(II) to the anammox bacteria. Zhen et.al.^33^ pointed out the heavy metal entering the sludge by active transport grow with the extending of exposure time. The accumulated heavy metal in anammox microorganisms cannot been removed by simple washing. Most of the anammox cells may be substantially damaged when the anammox bacteria exposed to Ni(II) for 48 h. These lead to a bad anammox activity recovery with 48 h the exposure time. The inhibition of anammox activity by Ni(II) with exposure time no more than 24 h can be eliminated after EDTA washing.

### Mechanism analysis of the effects of Ni(II) on anammox

#### Relationship between SAA and HDH activity

**Figure 5:**
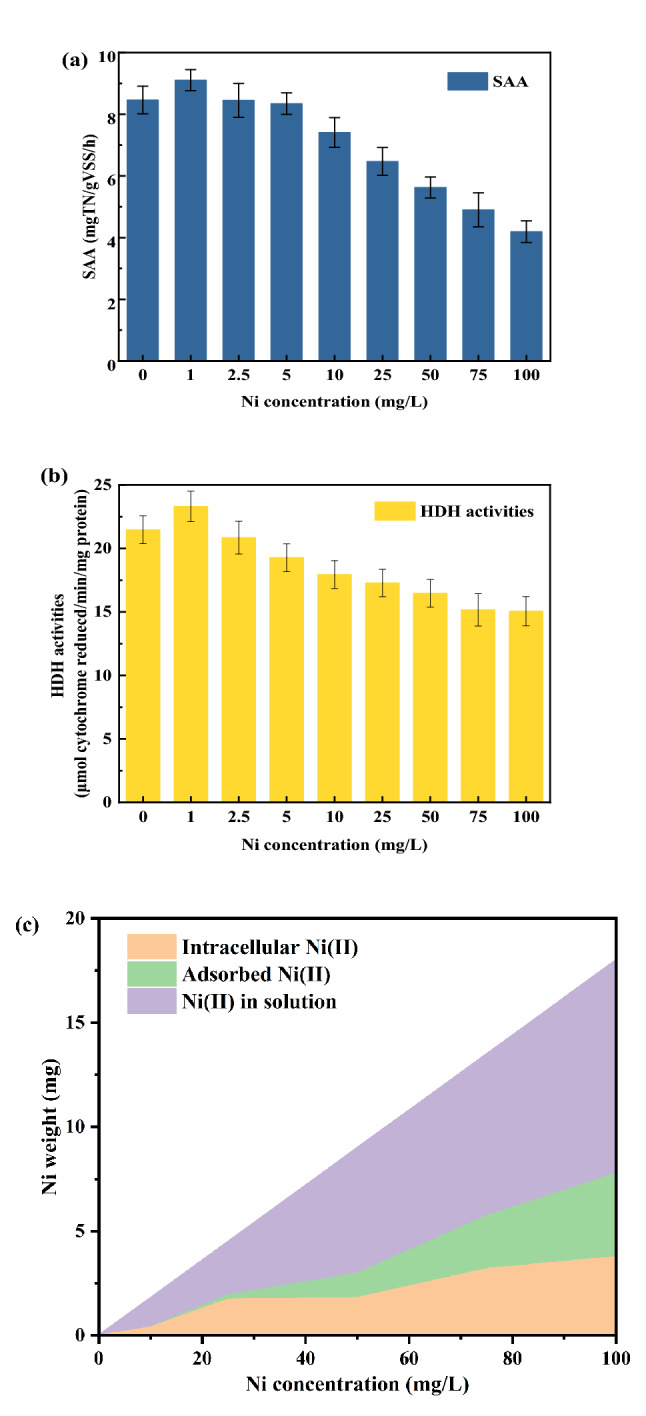
SAA values (**a**), HDH activity (**b**) and Ni(II) distribution in different regions (**c**) in batch experiments with different Ni(II) addition.

As shown in Fig. 5a, the SAA significantly increased from 8.46 to 9.11 mg-TN/g-VSS/h with Ni(II) concentration increasing from 0 to 1 mg/L (*p* < 0.05). The SAA kept falling with a further increasing of Ni(II) concentration. The SAA with 1 mg/L Ni(II) addition was enhanced by 11.14% and 8.56% than that without Ni(II) addition. The promoting effect of low concentration of metal on the anammox activity has also been reported in the previous study. Li et al.^31^ reported that nitrogen removal rate and SAA increased by 14.64% and 57.88%, respectively, with Ni(II) addition (1.5 mg/L).

Similar change trend was appear between HDH activity and Ni(II) concentration (Fig. 5b). The HDH activity significantly increased from 21.6 µmol-cytochrome reduecd/min/mg-protein to 23.7 µmol-cytochrome reduecd/min/mg-protein with Ni(II) concentration increasing from 0 mg/L to 1 mg/L (*p* < 0.05), and then decreased with a further increasing of Ni(II) concentration. The HDH activity with 1 mg/L Ni(II) addition were improved by 8.56% than that without Ni(II) addition. HDH is the most important enzyme in the anammox process, and can convert the intermediate hydrazine (N_2_H_2_) into nitrogen^34,35^. Ma et al.^36^ reported substrate degradation refers to the consumption of substrate to generate more energy with the exposure of Zn(II) under the guidance of energy-harvesting-related mRNAs (nitrite reductase and hydrazine oxidation). The corresponding HDH in anammox reaction systems increased when heavy metals addition with a low concentration. Once its concentration exceeds a threshold, heavy metals will inhibit the growth of the microorganism either sustainably or reversibly^37^. The enhancement of HDH activity with 1 mg/L Ni(II) addition accelerated the reaction of nitrogen conversion. Ni(II) addition with low concentration may promoted anammox activity and resulted in more nitrogen removal by anammox bacteria to resist Ni(II) toxicity. 1 mg/L Ni(II) addition improved the nitrogen removal performance of anammox sludges. Anammox activity was inhibited and HDH activity decreased with Ni(II) addition ≥ 2.5 mg/L.

#### Relationship between the distribution of Ni(II) and SAA

Ni(II) existed mainly in the forms of soluble Ni(II), extracellular adsorption Ni(II) and intracellular accumulation Ni(II) in our reaction systems. The content of Ni(II) distributed to different three regions in sludges all increased with a raise of Ni(II) addition (Fig. 5c). When 25 mg/L Ni(II) was added, the content of intracellular accumulation Ni(II) was about 1.728 mg. After then, the intracellular accumulation Ni(II) content rapidly growed with a further increasing of Ni(II) addition. Intracellular accumulation Ni(II) content was very little in the reaction systems with Ni(II) addition no more than 25 mg/L. When Ni(II) concentration was no more than 25 mg/L, nitrogen removal was mainly attributed to anammox reaction and higher NH_4_^+^-N removal efficiency (83%-99%) and NO_2_^−^-N removal efficiency (94%-95%) were gotten in our experiments (Figs. 1 and 3). The different experimental phenomenon appeared with a further increasing of Ni(II) concentration. This indicated that the increasing of intracellular accumulation Ni(II) may cause the change of nitrogen removal by the anammox sludges with more than 25 mg/L Ni(II) addition. Hence, the anammox activity inhibited by Ni(II) with more than 25 mg/L addition may be mainly brought by intracellular accumulation Ni(II).

To further clarify the mechanism of Ni(II) inhibition on anammox bacteria, the relation of intracellular accumulation Ni(II) and SAA inhibition percent was investigated and summarized in Table 2. A high R^2^ of 0.9850 was gotten by using a biotoxicity model (Eq. 3) to simulate the relation of intracellular accumulation Ni(II) and SAA inhibition percent. The biotoxicity model was suitable to describe the relation of intracellular accumulation Ni(II) and SAA inhibition percent. This further exposes SAA inhibition by Ni(II) was more closely related to the intracellular accumulation.

Ni(II) ingested by microorganisms can disrupt protein with functional groups. In general, the toxicity of heavy metals to biological systems is due to the effect of heavy metals on enzyme and protein functions. Although Ni(II) is a trace element required for the growth of anammox bacterial, excessive Ni(II) in anammox bacterial damaged protein structure and reduced activity of HDH, resulting in the deterioration of nitrogen removal performance. When excessive Ni(II) was present in the batch experiments, the Ni(II) content of intracellular accumulation was the dominant factor for SAA inhibition.

### Cumulative effect

**Figure 6:**
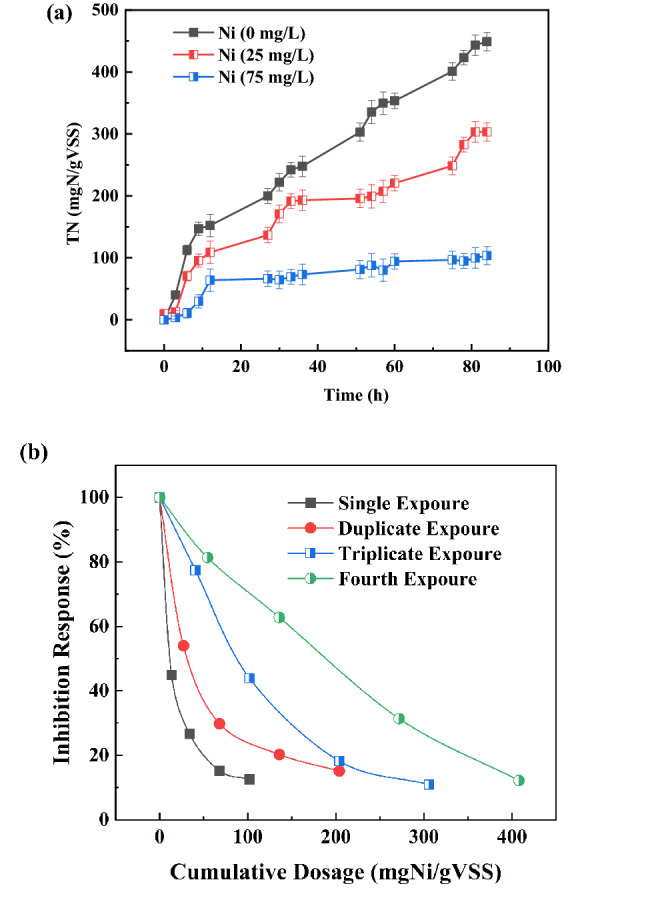
The cumulative TN removal with different Ni(II) addition (**a**) and the inhibition response to Ni(II) accumulated in the sludges with 25 mg/L Ni(II) addition (**b**).

Experiments with four different cycles were carried out to explored the bioaccumulation effect of Ni(II) on the anammox sludges. Each cycle was about 24 h, including 12 h experimental phase and 12 h intermittent phase. As shown in Fig. 6a, TN cumulative removal without Ni(II) addition was 449 mg-TN/g-VSS after four times cycles. Corresponding, the TN cumulative removal with 25 mg/L Ni(II) and 75 mg/L Ni(II) addition were about 303 mg-TN/g-VSS and 103 mg-TN/g-VSS, respectively. When Ni(II) addition was 25 mg/L, the TN cumulative removal after four times cycles obviously increased than that after three times cycles. When Ni(II) addition was 75 mg/L, there was no significant difference about TN cumulative removal between three times cycles and four times cycles. And, the TN cumulative removal with 75 mg/L Ni(II) addition was only 31.57% of that without Ni(II) addition. The inhibition of anammox microorganisms by Ni(II) addition was reduced after multiple cycles domestication with 25 mg/L Ni(II) addition. With a higher Ni(II) addition (75 mg/L), the inhibition did not eliminate well with multiple cycles domestication.

The inhibition response to Ni(II) accumulated in the sludges with 25 mg/L Ni(II) addition was shown in Fig. 6b. The relation between the inhibition response and Ni(II) concentration of the anammox sludges with different times cycles were exponentially fitted by (Eq. 2). The fitting equations, fitting coefficient R^2^ and IC_50_ calculated by (Eq. 2) were summarized and listed in Table 3. The IC_50_ values for one-, two-, three- and four-times cycles were 10.83, 30.89, 82.08, and 165.43 mg/L, respectively. The IC_50_ values increased with a raise in the number of cycles. Therefore, with low Ni(II) concentration (≤ 25 mg/L) addition, anammox microorganisms can extend their tolerance to Ni(II) by multiple intermittent exposures to Ni(II).

**Table 3:** IC_50_ determination with different domestication cycles.

Domestication cycles	Inhibition regression equation	R^2^	IC_50_
1	$IP = 100 \times \left( {\frac{1}{{1 + \left( {\frac{c}{10.83871}} \right)^{0.89781} }}} \right)$	0.99968	10.83
2	$IP = 100 \times \left( {\frac{1}{{1 + \left( {\frac{c}{30.89777}} \right)^{0.95712} }}} \right)$	0.9985	30.89
3	$IP = 100 \times \left( {\frac{1}{{1 + \left( {\frac{c}{82.08358}} \right)^{1.68673} }}} \right)$	0.9993	82.08
4	$IP = 100 \times \left( {\frac{1}{{1 + \left( {\frac{c}{165.43606}} \right)^{1.72543} }}} \right)$	0.98375	165.43

### Recovery of anammox activity after Ni(II) shock

**Figure 7:**
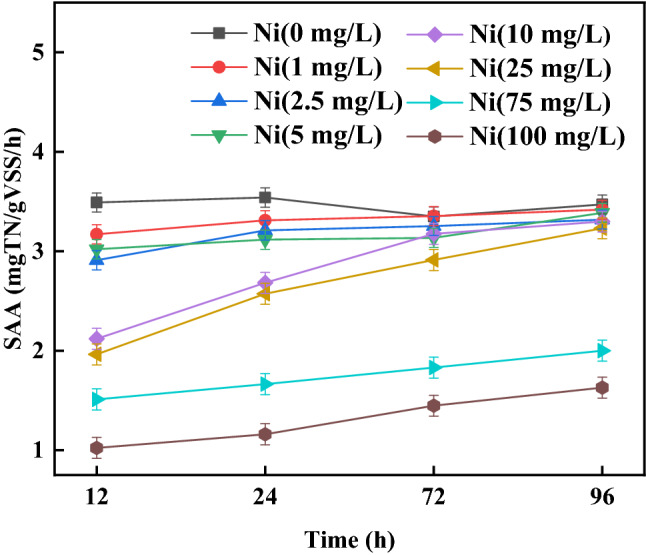
The activity recovery of anammox sludge with different Ni(II) addition.

Recovery of anammox activity of the anammox sludges with different Ni(II) addition were presented in Fig. 7. The sludges exposed to different Ni(II) concentration were washed by EDTA washing. When Ni(II) concentrations were 2.5, 5, 10, and 25 mg/L, the SAA recovered to 98.49%, 95.61%, 97.42%, and 93.25% of the SAA without Ni(II) addition. When Ni(II) concentrations were 75 and 100 mg/L, only 53.47% and 46.49% of the SAA without Ni(II) addition were gotten after EDTA washing, respectively. When Ni(II) concentration was no more than 25 mg/L, Ni(II) not in liquid most stayed outside of the sludges through extracellular adsorption (Fig. 5c). Little Ni(II) entered into the inside of the sludges with Ni(II) addition no more than 25 mg/L. The inhibition of anammox activity by Ni(II) with lower Ni(II) addition can be eliminated after EDTA washing. However, Ni(II) content of intracellular accumulation increased with a further raise of Ni(II) concentration. High Ni(II) concentration may lead to an irreversible deactivation of the anammox bacteria. Intracellular accumulation Ni(II) is not easy to clean by EDTA washing. A poor recovery of anammox activity was gotten by EDTA washing for the anammox sludges with Ni(II) addition higher than 25 mg/L.

## Conclusion

Anammox was the dominant reaction in batch experiments with Ni(II) addition no more than 25 mg/L. Ni(II) addition with low concentration (1 mg/L) may stimulate electron transfer processes of anammox and promoted anammox activity, resulting in a more nitrogen removal by anammox bacteria. High Ni(II) concentration and more exposure time resulted in the low nitrogen removal performance of the anammox sludges. When Ni(II) addition was higher than 25 mg/L, anammox activity was inhibited and HDH activity decreased. Intracellular accumulation Ni(II) was the dominant factor for anammox activity inhibition. Multiple cycles domestication and EDTA washing can eliminate the inhibition of anammox activity by Ni(II) with Ni(II) addition no more than 25 mg/L.

## Data Availability

The datasets generated during and/or analyzed during the current study are available from the corresponding author on reasonable request.
